# Functional Studies and Expression Characteristics of the Vacuolar Sugar Transporter CoSWEET2a in *Camellia oleifera*

**DOI:** 10.3390/plants14172618

**Published:** 2025-08-22

**Authors:** Xinhui Zou, Bingshuai Du, Jing Zhou, Jingjing Hu, Yibo Cao, Lingyun Zhang

**Affiliations:** 1 Key Laboratory of Forest Silviculture and Conservation of the Ministry of Education, The College of Forestry, Beijing Forestry University, Beijing 100083, China; zouxinhui89@163.com (X.Z.); dubingshuai624@163.com (B.D.); zhoujin.1234@163.com (J.Z.); hujingjing7787@163.com (J.H.); caoyibo@bjfu.edu.cn (Y.C.); 2 Beijing Engineering Research Center for Deciduous Fruit Trees, Key Laboratory of Biology and Genetic Improvement of Horticultural Crops (North China), Ministry of Agriculture and Rural Affairs, Institute of Forestry and Pomology, Beijing Academy of Agriculture and Forestry Sciences, Beijing 100093, China

**Keywords:** *Camellia oleifera*, SWEET transporter, sugar transport, hormone response, drought stress

## Abstract

Sugar transporters of the SWEET family are essential for plant growth, development, yield formation, and stress responses by regulating sugar transport and distribution. This study characterizes the function and expression characteristics of CoSWEET2a, a Clade I SWEET gene in *Camellia oleifera*. We conducted subcellular localization, functional complementation in *Arabidopsis*, sugar response assays, drought tolerance tests, and hormone induction analysis. A key finding is *CoSWEET2a*, which that is localized on the vacuolar membrane in *Camellia oleifera*. Heterologous expression in *Arabidopsis atsweet2* mutants revealed sugar-specific effects on root growth. Moreover, expression of *CoSWEET2a* increased soluble sugar content in *Arabidopsis* seeds. Additionally, *CoSWEET2a* overexpression enhanced drought stress tolerance by augmenting sugar content. The expression of *CoSWEET2a* is regulated by gibberellin (GA) and abscisic acid (ABA), and its promoter contains corresponding hormone response elements. In conclusion, CoSWEET2a functions as a “sugar buffer” on the vacuolar membrane, regulating sugar accumulation, root development, and drought stress responses. This discovery not only reveals that vacuolar SWEET plays an important role in maintaining cytoplasmic sugar homeostasis in plants but also provides a direct genetic target for engineering high-quality, drought-tolerant crops.

## 1. Introduction

Sugar is a fundamental component in plant energy metabolism, acting as the principal form of photosynthetic products and a key mediator in cell osmoregulation and signaling [1]. The transport of photosynthetic products in plants follows a three-stage process: (1) phloem loading in the leaves, (2) long-distance transport within the sieve element–companion cell complex (SE/CC), and (3) phloem unloading in sink organs [2]. This unloading process takes place through symplasmic transport, the apoplasmic pathway, or a combination of the two, with the apoplasmic pathway relying on sugar transporter proteins to facilitate sugar transmembrane transit [3].

The SWEET (Sugars Will Eventually be Exported Transporters) family has received a lot of attention for its bidirectional transport capabilities [4,5,6], playing vital roles in plant growth, development, yield formation, and responses to biotic and abiotic stresses by regulating sugar allocation [7,8]. A typical SWEET protein consists of seven transmembrane helices. These helices connect two MtN3/saliva structural domains. SWEET proteins allow substrates like as sugars and hormones to be transported across membranes based on concentration gradients [9]. Phylogenetic analysis divided the SWEET family into four subfamilies: Clades I-IV. Clade I is principally responsible for hexose transfer. Proteins that transport glucose include AtSWEET2 [10], OsSWEET2b [11], and StSWEET1g [12]. Critically, several SWEETs localized to the vacuolar membrane have been implicated in stress tolerance mechanisms. *AtSWEET2* limits the accumulation of glucose in vacuoles, which imparts resistance to pathogens [10]. *AtSWEET17* reduces drought stress by encouraging the development of lateral roots through vacuolar fructose efflux [13]. Moreover, germination and cold tolerance are improved by overexpressing *AtSWEET16*, which is located in the vacuolar membrane [14]. In woody plants, *CsSWEET16* overexpression in *Arabidopsis* decreases fructose buildup leaves at low temperatures, indicating that it plays a role in the vacuolar fructose efflux pathway [15]. These findings underscore the significance of vacuolar membrane-localized SWEETs in plant adaptation.

*Camellia (C.) oleifera* Abel. is a member of the *Camellia* genus and is mostly found in subtropical areas [16]. The seeds of *C. oleifera* are rich in oil, with high-quality content and a significant proportion of unsaturated fatty acids [17]. These seeds can be converted into edible oil, which has enormous potential [18]. Droughts in the summer and fall have become increasingly common in recent years due to global warming and abnormalities in air circulation within the primary oil tea production areas [19]. *C. oleifera* grows in hilly areas where artificial irrigation is difficult. The drought stress reduces the photosynthetic rate, number of flower buds, fruit set rate, fruit weight, and seed oil content of *C. oleifera*, significantly lowering the yield and economic benefits of *C. oleifera* orchards [20,21,22]. Sugar enhances the plant’s tolerance to stress at the levels of physiological homeostasis and molecular pathways by acting as a signaling switch and an osmotic pressure buffer [23]. Therefore, it is imperative to look into the mechanisms behind the development and stress tolerance of *C. oleifera* in order to cultivate high-yielding, drought-resistant types that will assure the industry’s sustained growth.

Sugar transport to developing seeds involves complex apoplast–plasmodesmata transitions, where vacuolar sugar homeostasis in transport cells plays a balancing role [24]. It is thought that CoSWEET2a, currently the only SWEET protein found in *C. oleifera* that is located in the vacuolar membrane, is essential to this process [24]. *CoSWEET2a* is highly expressed in seeds and responds to drought stress [25], suggesting that it may perform a similar function in stress resistance as the characterized vacuolar Clade I SWEET proteins. Additionally, vacuoles play a key role in material storage and homeostasis regulation [26]. Therefore, elucidating the molecular function and physiological mechanisms of CoSWEET2a not only holds promise for revealing its specific contributions in *C. oleifera* but also offers new insights into how woody perennial plants optimize energy storage and respond to environmental challenges such as drought through adaptive sugar transport mechanisms.

In this work, we examined the protein structure and oligomerization characteristics of CoSWEET2a. We directly localized CoSWEET2a to the vacuolar membrane using mesophyll protoplasts from the woody oil crop *C. oleifera*. This represents the first structural validation of tonoplast targeting for a SWEET transporter in a perennial oil plant. When *CoSWEET2a* was overexpressed in *Arabidopsis*, the level of soluble sugar in the seeds increased. *CoSWEET2a* also affected the growth of root length under treatments with sucrose, glucose, and fructose. It also increased seedling survival under drought stress, as well as an overall increase in soluble sugar content throughout the plant. We established a causal chain linking CoSWEET2a-mediated vacuolar sugar buffering to systemic sugar accumulation and drought resilience. GA and ABA increase the expression of *CoSWEET2a*, providing evidence for hormonal regulation of vacuolar SWEET activity. As a “vacuolar sugar buffer” for hormone signal response, *CoSWEET2a* is critical in coordinating seed sugar buildup, root development, and drought tolerance.

## 2. Results

### 2.1. CoSWEET2a Is Localized to the Vacuolar Membrane in C. oleifera

**Figure 1:**
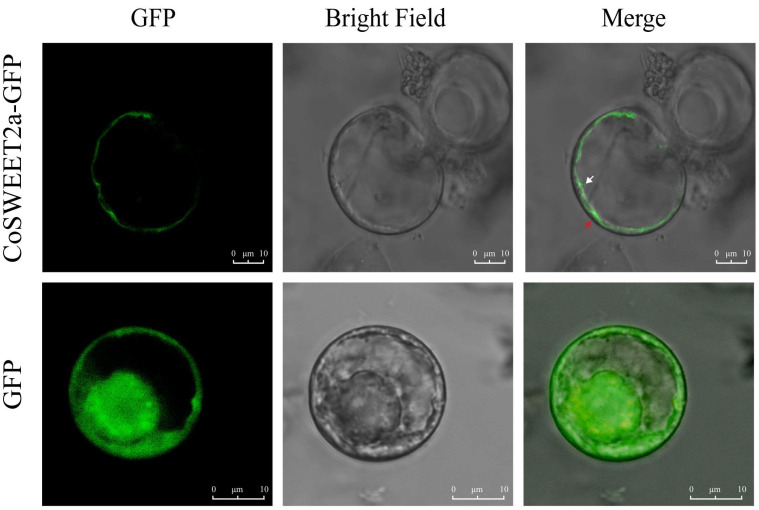
The localization of CoSWEET2a in protoplasts of *C. oleifera*. White arrows point to the vacuolar membrane, and red arrows mark the plasma membrane. The empty vector (GFP) was a positive control. Scale bar = 10 μm. The experiment was conducted independently three times, and three images were captured each time.

Through heterologous transient transformation in tobacco (*Nicotiana benthamiana*), Du et al. [24] discovered that the CoSWEET2a protein is localized to vacuolar membranes. To precisely determine the subcellular localization within *C*. *oleifera* tissues, we performed transient transformation in protoplasts derived from callus and examined the subcellular localization of *CoSWEET2a*. Confocal microscopy revealed that CoSWEET2a-GFP fluorescence exhibited an invaginated structure distally located from the nucleus (Figure 1), indicating consistency with the characteristic morphology of the tonoplast. However, in the control group (transformed with an empty vector), fluorescent signals were diffusely distributed throughout the cell of leaves. These findings support CoSWEET2a’s location in the vacuolar membrane.

### 2.2. Basic Characteristics and Phylogenetic Analysis of CoSWEET2a

**Figure 2:**
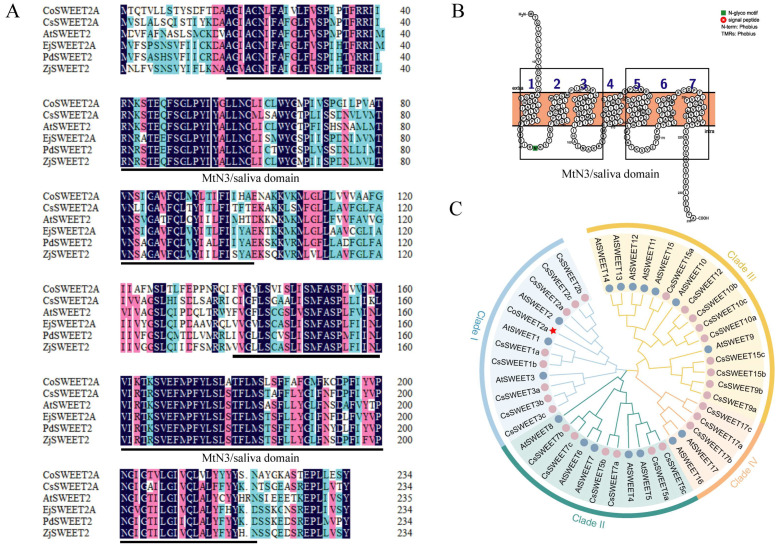
Bioinformatics analysis of *CoSWEET2a*. (**A**) Multiple sequence alignment of the CoSWEET2a amino acid sequence with homologous sequences from other plants. The two conserved MtN3_slv domains are indicated by black lines. (**B**) Transmembrane domains of CoSWEET2a. The seven transmembrane domains (TMD1–TMD7) are numbered sequentially from the N-terminus to the C-terminus. The MtN3_slv domains are highlighted with black boxes. (**C**) Phylogenetic analysis of CoSWEET2a with proteins from other species. CoSWEET2a is highlighted in red star. *Arabidopsis thaliana* is marked with a blue circle, and *Camellia sinensis* is marked with a red circle.

The CoSWEET2a gene’s coding sequence (CDS) spans 708 nucleotides and codes for a 235 amino acid protein (Tables S1 and S2). This protein has a molecular mass of 25.99 kDa, with an average hydrophilicity index of 0.926, indicating that it is extremely hydrophobic and may be involved in membrane-associated processes (Figure S1A). By aligning the CoSWEET2a’s sequence with the NCBI database to find homologous SWEET proteins in order to categorize it and forecast its possible function (Table S2). According to sequencing research, CoSWEET2a and its related proteins have a significant degree of conservation, especially in the amino acid sequence areas 18–100 and 138–220. These areas match the SWEET family’s distinctive MtN3/sativa domain (Figure 2A), which is made up of three transmembrane domains (TMs) at the front and back (Figure 2B). Additionally, the transmembrane domains correspond to the tertiary (Figure S1B). Additionally, a phylogenetic tree comparing CoSWEET2a with members of the SWEET family from *A. thaliana* and *C. sinensis* was created using the maximum likelihood method (Table S2). The findings showed that AtSWEET2 and CsSWEET2a/b/c, both of which are members of Clade I, are the most closely related to CoSWEET2a (Figure 2C).

### 2.3. CoSWEET2a Forms Homodimers at the Vesicular Membrane

**Figure 3:**
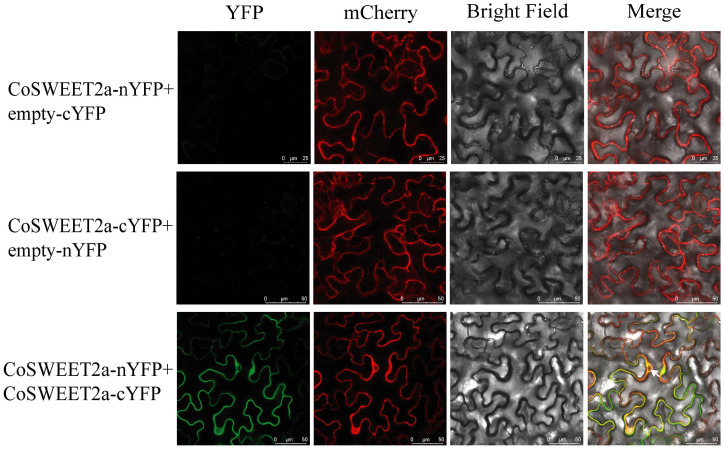
Bimolecular fluorescence complementation analysis of CoSWEET2a self-interaction. The green signals indicate YFP, whereas the red signals indicate the plasma membrane marker vac-CD3-971 vector (mCherry). The yellow signals represent co-localization of YFP and mCherry signals. White arrowheads indicate the cell nucleus. The experiment was conducted independently three times, and three images were captured each sample.

Prior research has shown that some SWEET proteins can increase the pace or efficiency of macromolecular sugar transport by forming homo- or heterodimers with other transporters or with themselves [27]. We used a bimolecular fluorescence complementation (BiFC) assay for insight into the transport form of CoSWEET2a in plants. In this experiment, *CoSWEET2a* was fused to the N-terminal and C-terminal ends of YFP proteins, respectively, and then transformed into tobacco epidermal cells. YFP fluorescence localized to the vacuolar membrane was clearly evident when CoSWEET2a-cYFP and CoSWEET2a-nYFP were co-transformed, according to fluorescence observation. This fluorescence overlapped with the red fluorescence of the vacuolar membrane marker vac-CD3-971. Notably, neither vac-CD3-971 [28] nor YFP fluorescence went to the nucleus (Figure 3). However, no green fluorescence was seen in either scenario when CoSWEET2a-cYFP and CoSWEET2a-nYFP were injected separately. These findings imply that CoSWEET2a can form a homodimer with itself at the vacuolar membrane.

### 2.4. CoSWEET2a Overexpression Enhances Sugar Accumulation in Seeds

To investigate the function of *CoSWEET2a* and its impact on plant growth, development, seed yield, and other aspects, we generated the homozygous *atsweet2* mutant in *Arabidopsis*. After that, the *35S::CoSWEET2a* overexpression vector was constructed and introduced into the mutants. After identification by PCR and RT-qPCR, two homozygous restoration lines, *CoSWEET2a*-R6 and *CoSWEET2a*-R8 were selected for further studies (Figure S2).

**Figure 4:**
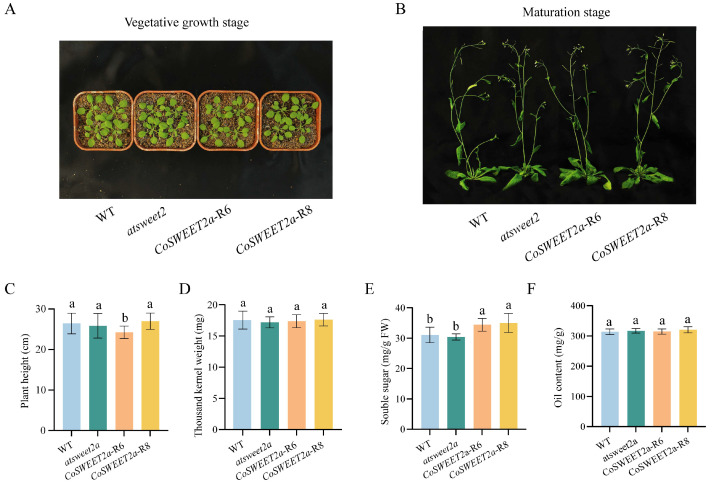
Phenotypic and physiological characterization of *Arabidopsis* transgenic lines. (**A**,**B**) Phenotypes of *Arabidopsis* wild type (WT), *atsweet2* mutants, and restoration lines (*CoSWEET2a*-R6, and (*CoSWEET2a*-R8) during vegetative growth stage (**A**) and maturation stage (**B**). (**C**) Measurement of plant height in mature *Arabidopsis* plants. (**D**–**F**) Statistics of thousand-seed weight (**D**), determination of sugar content (**E**), and determination of oil content (**F**) in seeds from different *Arabidopsis* lines. Different letters indicate significant differences between groups (*p* < 0.05), while the same letters indicate no significant differences (*p* > 0.05). Three independent biological replicates were used for the experiment.

*Arabidopsis* wild-type, *atsweet2* mutant, and *CoSWEET2a* restoration lines were germinated and cultivated under the same circumstances. During the vegetative and maturation stages, there were no discernible variations in the lines’ growth (Figure 4A,B). R6’s average height at maturity (24.22 cm) was much shorter than that of the other strains. Compared to the wild-type, mutant, and R8, the decrease was roughly 8.3%, 6.2%, and 10.1% (Figure 4C). We also took measurements of the sugar content and seed production. Neither the oil content nor the weight of the thousand seeds changed significantly (Figure 4D,F). In contrast, the sugar content of the restoration lines was 14.14% higher on average than that of the wild-type and mutant (Figure 4E). These data show that *CoSWEET2a* expression increased seed sugar content without markedly affecting plant growth or yield.

### 2.5. Analysis of Seedling Root Growth of Arabidopsis Lines Under Different Sugar Treatments

**Figure 5:**
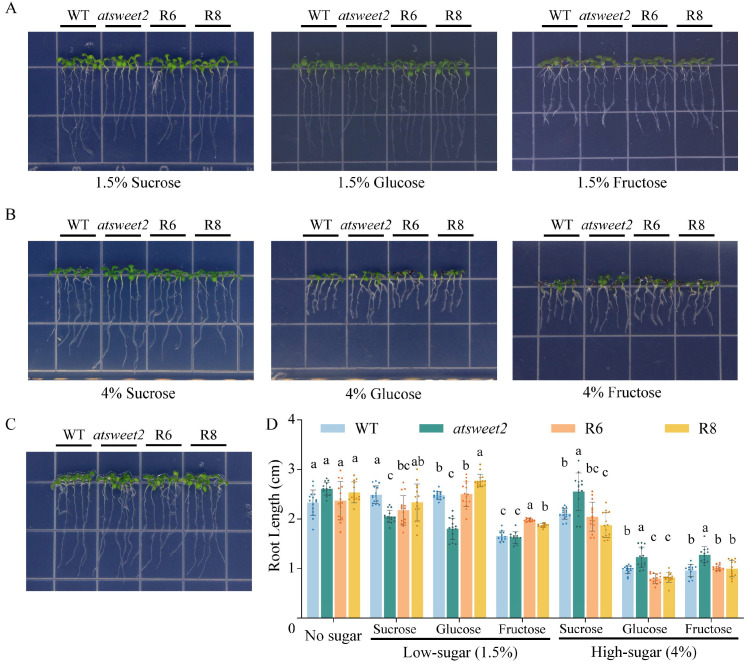
Phenotypic and root length analysis of *Arabidopsis* wild type (WT), *atsweet2* mutant, and two restoration lines (R6 and R8) under different sugar treatments. (**A**–**C**) Above-ground and root phenotypes of *Arabidopsis* treated with sugar free (no sugar) (**C**), sucrose, glucose, and fructose at concentrations of 1.5% (**A**) and 4% (**B**). (**D**) Main root length between *Arabidopsis* lines under different sugar treatments. The color circles represent individual data points. The seven treatments in the figure were analyzed independently using one-way ANOVA (α = 0.05); multiple comparisons were performed using Duncan’s method. Different letters indicate significant differences between groups (*p* < 0.05), while the same letters indicate no significant differences (*p* > 0.05). Three independent biological replicates were used for the experiment.

WT, *atsweet2* mutant, CoSWEET2a-R6, and CoSWEET2a-R8 seedlings were used as study materials to examine how *CoSWEET2a* responds to various sugar types in plants. The seedlings were vertically cultured for one week on sugar free (no sugar), low-sugar (1.5%), and high-sugar (4%) sucrose, glucose, and fructose media, respectively. Under low-sugar conditions (Figure 5A), *atsweet2* showed a significant root length shortening phenotype, which could be rescued by CoSWEET2a. Compared to the WT, the restoration lines (R6, R8) showed an average increase of 16.1% in root length under fructose treatment. Both WT and the restoration lines’ root length was reduced to varying degrees under high-sugar stress (Figure 5B). In contrast, the mutant exhibited minimal inhibition, suggesting that *AtSWEET2* deletion may lessen the effects of high-sugar stress. The greatest root length shortening in R6 and R8 was observed under 4% glucose conditions, suggesting that *CoSWEET2a* heterologous expression enhances high sugar stress and encourages glucose translocation. To sum up, the CoSWEET2a protein facilitates the uptake of hexose (glucose and fructose) and exhibits a response to sucrose.

### 2.6. Phenotypic Observation and Analysis of Arabidopsis Strains Under Drought Treatment

**Figure 6:**
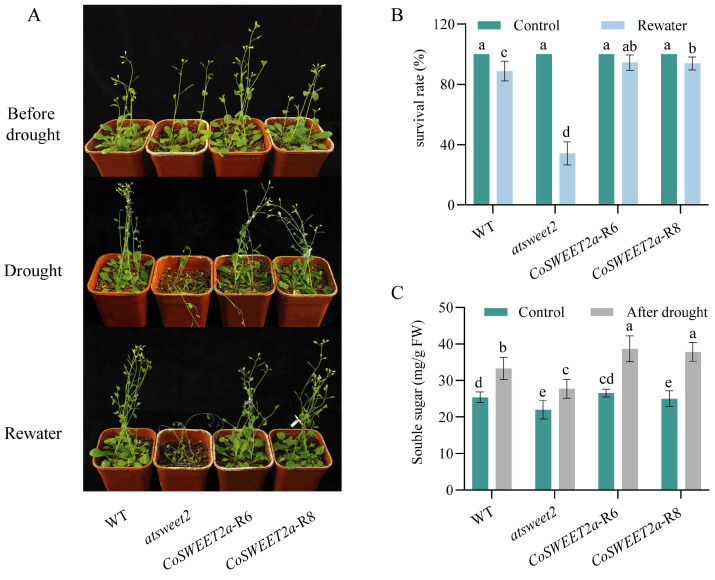
Phenotypic analysis of transgenic *Arabidopsis* under drought stress. (**A**) Phenotypic responses of *Arabidopsis* lines to drought treatment. Drought treatment for 14 days followed by rehydration for 5 days. (**B**) Survival rate of *Arabidopsis* seedlings on the fifth day of rehydration. Control is untreated group. Plants were considered survivors if their leaves turned green again or if new green leaves emerged. (**C**) The soluble sugar contents of WT, *atsweet2*, *CoSWEET2*-R6, and *CoSWEET2*-R8 in whole *Arabidopsis* plants. Different letters indicate significant differences between groups (*p* < 0.05), while the same letters indicate no significant differences (*p* > 0.05). Three independent biological replicates were used for the experiment.

We evaluated drought resistance in several *Arabidopsis* lines for insight into how *CoSWEET2a* affects the plant’s reaction to drought stress. Drought treatment was applied to four-week-old *Arabidopsis* seedlings for around two weeks. The *atsweet2* mutant’s leaves were severely wilting (Figure 6A), and a reduced survival after recovery (Figure 6B). These findings imply that *CoSWEET2a* considerably improves plants’ ability to survive drought.

Soluble sugar levels were measured in drought-treated and untreated plants across different lines (Figure 6C). Under drought, all *Arabidopsis* lines showed a considerable increase in soluble sugar content. Interestingly, the *CoSWEET2a* restoration lines showed greater increases (~45–51%) compared to wild-type (31.1%) and mutant (26.1%) lines.

### 2.7. Analysis of CoSWEET2a Gene Promoter Elements and Response to Different Hormones

**Figure 7:**
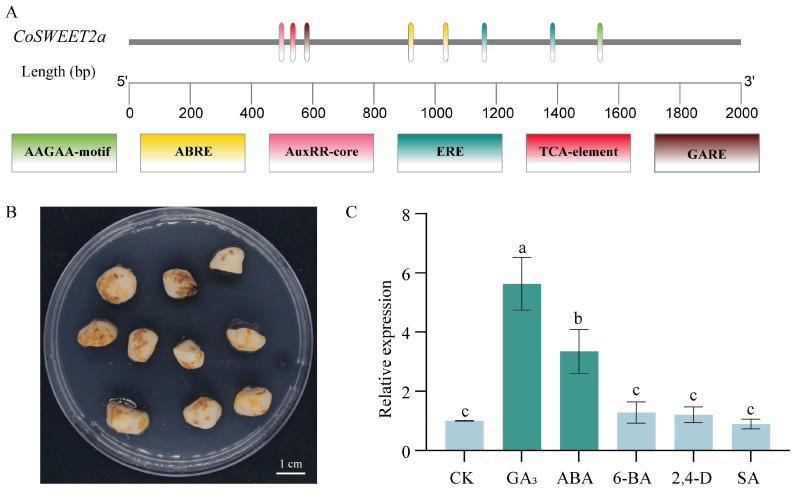
Promoter element analysis of *CoSWEET2a* and expression of *CoSWEET2a* under different hormone treatments. (**A**) Hormone-responsive elements in the *CoSWEET2a* promoter sequence. (**B**) Immature *C. oleifera* seeds cultured in vitro. (**C**) Expression patterns of *CoSWEET2a* under different hormone treatments: GA_3_ (Gibberellin), ABA (Abscisic acid), 2,4-D (Auxin), and SA (Salicylic acid). Different letters indicate significant differences between groups (*p* < 0.05), while the same letters indicate no significant differences (*p* > 0.05). Three independent biological replicates were used for the experiment.

Plants react to abiotic stressors through a variety of intricate processes, such as hormone synthesis, ROS scavenging, stress-responsive gene expression, and other tactics. By controlling downstream target genes, phytohormone signaling is one of them that can coordinate the stress response. This study first examined the upstream 2000 bp sequence of the *CoSWEET2a* gene. In addition to the fundamental elements, this region has four kinds of cis-acting elements (Figure S3). These include hormone-responsive elements like gibberellin response element (GARE motif), salicylic acid-responsive element (TCA-element) and abscisic acid-responsive elements (ABRE and AAGAA-motif), as well as regulatory elements for the abiotic stress response like the wound-responsive element (WUN-motif) (Figure 7A).

By providing exogenous hormones to immature oil tea seeds in vitro, the regulatory mechanism was further confirmed based on the aforementioned predictions (Figure 7B). After eight days of dark incubation, it was found that the *CoSWEET2a* gene was highly upregulated by both GA_3_ and ABA, with relative expression increasing by around six and three times, respectively. The change in expression was not significantly impacted by treatments like SA and IAA (Figure 7C).

## 3. Discussion

SWEET family proteins are found in both prokaryotes and eukaryotes, and they are essential for organ development, stress responses, and photosynthetic assimilate partitioning [29,30]. According to prior research, CoSWEET2a is localized to the vacuolar membrane in tobacco epidermal cells and protoplasts [24], but is found throughout the yeast cell [24]. This species-specific localization pattern motivated us to do the first subcellular localization investigation of CoSWEET2a in the native C. oleifera system. Our results confirmed the vacuolar localization of *CoSWEET2a* (Figure 1), which is consistent with the results of previous studies on homologous genes, such as AtSWEET2 [31] and OsSWEET2b [11]. These results imply that the location of the vacuolar membrane may be a characteristic shared by some SWEET2 members in different plant species.

According to phylogenetic analysis, CoSWEET2a is a member of Clade I (Figure 2C). The primary function of SWEET proteins in Clade I is glucose transport [32]. However, yeast assays revealed CoSWEET2a transports glucose, sucrose, and fructose [24]. Our results confirm that *CoSWEET2a* responds to sucrose, fructose, and glucose in *Arabidopsis* (Figure 5A–C). This broad substrate specificity diverges from canonical Clade I functions and mirrors multisubstrate transporters like *Camellia sinensis* CsSWEET1a (Clade I), which transports hexoses and sucrose [15]. BiFC assay further indicates CoSWEET2a forms homodimers (Figure 3)—a feature previously linked to enhanced proteins’ stability and transport effectiveness [27]. Taken together, CoSWEET2a represents an evolutionarily distinct Clade I transporter with expanded substrate range, likely enabled by its homodimeric state for sucrose transport.

Traditional sink-strengthening SWEETs directly enhance yield traits. For instance, rice *OsSWEET4c* promotes endosperm starch storage [33], and soybean *GmSWEET10a/b* supports embryo sugar supply [5]. In *C. oleifera*, *CoSWEET10* plays a comparable role in seed development and oil accumulation [34]. By contrast, *CoSWEET2a* serves a distinct physiological role. Heterologous expression in *Arabidopsis* elevated seed sugar content without altering oil content or thousand-seed weight (Figure 4C–E), indicating its role in sugar translocation, but it had no discernible impact on plant growth or production.

*CoSWEET2a* is expressed in the transfer cell layer of *C. oleifera* seeds, according to in situ hybridization results [24]. And its subcellular localization to vacuolar membranes (Figure 1). We propose that *CoSWEET2a* might serve as a “vacuolar sugar buffer”. We speculate that it may regulate sugar transport within vacuoles in response to changes in inside the cell sugar concentrations. Vacuoles temporarily store extra sugars when their influx to plasma membrane surpasses the embryo’s capacity for utilization [25]. However, further experiments are needed to verify the transport properties of CoSWEET2a, such as in vitro verification using an African clawed frog oocyte expression system. In summary, *CoSWEET2a* demonstrates a decoupling of vacuolar sugar accumulation and biomass formation, representing a unique mechanism in sugar transport regulation.

Sugar enhances the plant’s tolerance to stress at the levels of physiological homeostasis and molecular pathways by acting as a signaling switch and an osmotic pressure buffer [30]. Under drought stress, the *CoSWEET2a* restoration lines showed high survival rates and elevated sugar content (Figure 6A–C), and the relative expression level of *CoSWEET2a* was upregulated in *C. oleifera* [25]. Drawing from prior studies, we postulate that *CoSWEET2a* could enhance drought resistance via two possible pathways: (1) CoSWEET2a contributes to the maintenance of cytoplasmic osmotic pressure during drought by exporting vacuolar sugar [35].This vacuolar buffering mechanism allows plants to tolerate greater fluctuations in sugar concentrations, which could be an adaptive strategy for woody plants in variable environments. (2) Vacuolar sugar transporters may prioritize the redistribution of stored sugars from vacuoles to critical regions, such as meristematic tissues, under drought stress, ensuring essential metabolic needs are met. This aligns with the hypothesis proposed for AtSWEET17 [13,36]. These results confirm that *CoSWEET2a* contributes to drought tolerance by regulating sugar homeostasis, consistent with its “vacuolar sugar buffer” strategy for sugar dynamic balance. *CoSWEET2a* represents a promising genomic target for developing drought-resistant *C. oleifera* cultivars through precision breeding. However, its long juvenile phase and tetraploid genome complicate transgenic approaches. We recommend marker-assisted selection of natural variants using CAPS/dCAPS markers as a faster alternative to develop stress-tolerant varieties.

Phytohormonal signaling pathways, which alter target genes involved in drought adaptation, are commonly used by plants to control stress responses [37]. In this study, gibberellin and abscisic acid significantly increased the expression of *CoSWEET2a* in *C. oleifera* seeds treated with exogenous hormones (Figure 7C), consistent with their antagonistic roles in seed development [38]. We speculate that drought stress causes ABA accumulation, which in turn triggers *CoSWEET2a* expression. Several ABA-responsive bZIP transcription factors, such as rice *OsbZIP72* [39], apple *MdbZIP23/46* [40], have been shown to regulate SWEET gene expression. Since *CoSWEET2a* enhances drought resistance, identifying its regulators in *C. oleifera* could benefit breeding. Additionally, while certain SWEET family members (including *AtSWEET13/14* and *OsSWEET3a*) exhibit gibberellin transport activity [41,42], the capacity of *CoSWEET2a* to transport GA remains unconfirmed.

## 4. Materials and Methods

### 4.1. Plant Materials, Growth Conditions, and Stress Treatment

Fruit samples of *C. oleifera* ‘Huashuo’ were collected from the large-fruited oil tea test base in Changsha, Hunan Province. The fruits were harvested 230 days post-pollination (July), and the seeds were immediately frozen in liquid nitrogen and stored at −80 °C for subsequent RNA extraction. Concurrently, fruit samples with attached branches were collected and moisturized for seed culture experiments.

*Arabidopsis thaliana* and *Nicotiana benthamiana* were grown in an artificial climate chamber under a 16 h light/8 h dark cycle at 25 °C with approximately 60% relative humidity. Four-week-old *Arabidopsis* seedlings of similar growth status were subjected to drought treatment by water withholding for 14 days followed by 5 days of rewatering, with normal watered plants serving as controls. The drought treatment duration was referenced from Ye et al. [34]. For sugar treatment experiments, *Arabidopsis* seeds were sown on 1/2 MS medium and incubated in the dark at 4 °C. After germination, the seedlings were transferred to vertical culture plates containing different sugars and continued to grow for 5 days.

Callus tissue was induced from axillary buds of *C. oleifera* and cultured in dark conditions at 24 °C in a medium containing 4.42 g/L MS, 30 g/L sucrose, 5 g/L 2,4-D, and 6 g/L agar.

### 4.2. Cloning and Subcellular Localization of CoSWEET2a Gene

Total RNA was extracted from *C. oleifera* seeds using the Total RNA Extraction Kit for Polysaccharides and Polyphenols (Tiangen, Beijing, China). Reverse transcription was performed using the AccuRT gDNA Removal Kit (Applied Biological Materials, Richmond, BC, Canada). Gene-specific primers were designed with Primer Premier 5.0 (Premier Biosoft International, Palo Alto, CA, USA) (Table S3), and the target gene sequence was amplified using Primer STARMax DNA Polymerase (Takara Bio Inc., Kusatsu, Shiga, Japan), which offers high fidelity. The PCR products were separated by electrophoresis, and the target bands were subsequently recovered and ligated into the cloning T vector. The recombinant vector was then transformed into *E. coli* cells, and positive clones were selected using antibiotic resistance and sent for sequencing at Beijing Kinko Biotech.

The subcellular localization of CoSWEET2a was determined using an *C. oleifera* protoplast transient expression system. The CDS of *CoSWEET2a*, with the stop codon removed, was inserted into the overexpression vector pCAMBIA1300-GFP. Negative control was pCAMBIA1300-GFP empty vector. Protoplast isolation and PEG-mediated transformation were performed as described by Li et al. (2021, 2022) [43,44], except that the incubation time after transfection was shortened to 16 h in darkness. Fluorescence signals were observed with a Zeiss LSM 780 laser-confocal microscope (Carl Zeiss AG, Oberkochen, Germany) using the GFP channel (440–500 nm).

### 4.3. Bioinformatics Analysis

The sequence of the *CoSWEET2a* gene is provided in Supplementary Table S2. The physicochemical properties of the *CoSWEET2a* protein were analyzed using the Expasy server (https://web.expasy.org/ (accessed on 20 January 2025)). The tertiary structure of the *CoSWEET2a* protein was modeled using SWISS-MODEL (https://swissmodel.expasy.org/ (accessed on 20 January 2025)). Transmembrane structural domains were predicted using the Protter tool (http://wlab.ethz.ch/protter/start/ (accessed on 20 January 2025)). The homologous protein sequence of *CoSWEET2a* was retrieved from the NCBI database (https://www.ncbi.nlm.nih.gov/ (accessed on 20 January 2025)), and conserved structural domains were identified using the CDD website (https://www.ncbi.nlm.nih.gov/Structure/cdd/wrpsb.cgi (accessed on 20 January 2025)). Amino acid sequence comparisons were performed with DNAMAN version 9 (Lynnon Biosoft, San Ramon, CA, USA), and the phylogenetic tree was constructed using MEGA11 version 11.0.13 (Pennsylvania State University, PA, USA). The amino acid sequences employed for this phylogenetic analysis are listed in Supplementary Table S3. Sequences 2000 bp upstream of the *CoSWEET2a* transcriptional start site were extracted using TBtools v1.098 (https://github.com/CJ-Chen/TBtools), and the promoter region was analyzed for cis-acting elements using the online tools PlantCARE (https://bioinformatics.psb.ugent.be/webtools/plantcare/html/ (accessed on 20 January 2025)) and New PLACE (https://www.dna.affrc.go.jp/PLACE/?action=newplace (accessed on 20 January 2025)).

### 4.4. Bimolecular Fluorescence Complementation Experiments

The determination of the homodimerization ability of CoSWEET2a in plants was performed using tobacco leaf transient transformation technology. The CDS of *CoSWEET2a* was amplified without the stop codon and subsequently ligated into the vectors pSPYCE (cYFP) and pSPYNE (nYFP). The correctly sequenced CoSWEET2a-cYFP and CoSWEET2a-nYFP plasmids were transferred into *A. tumefaciens* GV3101. Tobacco epidermal cells were co-transfected with a vacuolar membrane localization marker (vac-CD3-971) [28]. According to the experimental setup described in Zhang et al. [45]. Negative controls consisted of CoSWEET2a-nYFP paired with empty-cYFP and empty-nYFP paired with CoSWEET2a-cYFP. After 36–48 h of transient expression, fluorescence signals were observed with a Zeiss LSM 780 laser-scanning confocal microscope (Carl Zeiss AG, Oberkochen, Germany) at 510 nm and 550 nm.

### 4.5. Generation of Arabidopsis Homozygous Mutants and Restoration Lines

Seeds of the *Arabidopsis atsweet2* mutant (SALK_034060) were obtained from the Arabidopsis Biological Resource Center (ABRC). The homozygous mutants were identified using a three-primer method: LP and RP primers were used to test the two insertion sites, while LBal was used to amplify the insertion fragment. Table S3 lists the primers that were used in this study. The recombinant *CoSWEET2a*-pCAMBIA1300 plasmid was transformed into Agrobacterium tumefaciens strain GV3101. The *atsweet2* mutant was then stably transformed using the inflorescence infiltration method [34]. After identifying heterozygous overexpression-positive plants by PCR and RT-qPCR, T3-generation purity restoration lines were selected and further screened using Timentin. Two homozygous restoration lines, *CoSWEET2a*-R6 and *CoSWEET2a*-R8, with high expression levels, were selected for further studies (Figure S2). Wild-type *Arabidopsis* plants served as controls.

### 4.6. Phenotypic Observation and Physiological Analysis of A. thaliana

In order to investigate the impact of *CoSWEET2a* on plant-related traits, a comprehensive phenotypic assessment was conducted on the *Arabidopsis* wild type (WT), *atsweet2* mutant, and two restoration lines (R6 and R8). The plant height was measured from the soil surface to the highest point of the plant at the time of seed maturity and recorded accordingly. One thousand seeds were randomly selected using the quadrat method, and their total weight was measured. The total soluble sugar content in the seeds was determined using anthrone colorimetry. The same method was applied to measure the sugar content in the whole plants of drought-treated *Arabidopsis*. The root length of *Arabidopsis* seedlings from each line was measured on culture plates.

### 4.7. In Vitro Culture of C. oleifera Seeds Under Different Hormone Treatments

To examine the effects of phytohormones on sugar metabolism-related gene expression during seed development, fruits of *C. oleifera* were harvested at 230 days after pollination (DAP), rinsed under running water, and disinfect in a sterile environment. Seeds with smooth, glossy epidermises and uniform sizes were selected and placed in culture medium for in vitro cultivation, with 10 seeds per medium. Seed culture conditions followed the protocol established by Sosso et al. [33], with minor modifications.

Seeds were cultured on modified MS medium (4.42 g/L MS, 1 g/L acid-hydrolyzed complex protein, 1 mg/L streptomycin, 30 g/L sucrose, and 5.8 g/L agar.) Experimental groups received basal medium supplemented with 1 μM dichlorophenoxyacetic acid (2,4-D), 1 μM gibberellic acid (GA_3_), 0.1 mM abscisic acid (ABA), and 10 mM salicylic acid (SA) in addition to the control medium. All cultures were incubated in the dark for 8 days.

### 4.8. RT-qPCR Analysis of CoSWEET2a Gene

To analyze the effect of hormone treatment on *CoSWEET2a* expression levels, RNA was extracted from *C. oleifera* seeds after 8 days of dark culture and reverse-transcribed into complementary DNA (cDNA) for subsequent RT-qPCR analysis, following the method outlined in Section 4.2. *CoActin* was used as the internal reference gene [46]. Primers for real-time quantitative PCR were designed as indicated in Table S1. The SYBR Green PCR Master Mix (Applied Biosystems, Foster City, CA, USA) was used as the reaction reagent, and quantitative reverse transcription PCR was conducted on the Step One Plus system, with an assay volume of 20 μL. The relative expression levels of *CoSWEET2a* were calculated using the 2^−∆∆CT^ method [47].

### 4.9. Data Processing

SPSS 27.0 software was used for statistical analysis. Significance was analyzed by one-way analysis of variance (ANOVA) and multiple comparisons test. Different letters indicate significant differences between groups (*p* < 0.05), while the same letters indicate no significant differences (*p* > 0.05). Data are expressed as the mean of at least 3 replications ± SD. At least five plants were used for each replicate. GraphPad Prism 9.0 and Adobe Illustrator 2020 were used for plotting.

## 5. Conclusions

*CoSWEET2a* acts as a “vacuolar sugar buffer”, integrating sugar transport and hormone response to improve drought tolerance. By simultaneously boosting seed soluble-sugar content and drought tolerance, *CoSWEET2a* exemplifies the critical role that vacuolar sugar transporters play when they modulate sugar homeostasis, a process which is vital for environmental adaptation. Our findings identify CoSWEET2a as a precise target for drought-tolerant transgenic breeding, as well as a direct candidate gene for marker-assisted natural variation selection.

## Data Availability

All relevant data can be found within the manuscript and its supporting material.

## Supplementary Materials

The following supporting information can be downloaded at https://www.mdpi.com/article/10.3390/plants14172618/s1, Table S1: Coding sequence (CDS) of *CoSWEET2a*, Table S2: Amino acid sequences used for homologous protein alignment and phylogenetic tree construction, Table S3: Sequences of primers used in this study; Figure S1: Hydrophilicity analysis (**A**) and tertiary structure prediction (**B**) of the CoSWEET2a protein, Figure S2: Identification of mutant and restoration lines of *Arabidopsis*, Figure S3: Analysis of cis-acting elements in the promoter region of the *CoSWEET2a* gene.

## References

[1] Lemoine R., La Camera S., Atanassova R., Dédaldéchamp F., Allario T., Pourtau N., Bonnemain J.L., Laloi M., Coutos-Thévenot P., Maurousset L. (2013). Source-to-Sink Transport of Sugar and Regulation by Environmental Factors. Front. Plant Sci..

[2] Ruan Y.L. (2014). Sucrose Metabolism: Gateway to Diverse Carbon Use and Sugar Signaling. Annu. Rev. Plant Biol..

[3] Ruan Y.L., Jin Y., Yang Y.J., Li G.J., Boyer J.S. (2010). Sugar Input, Metabolism, and Signaling Mediated by Invertase: Roles in Development, Yield Potential, and Response to Drought and Heat. Mol. Plant..

[4] Yang J., Luo D., Yang B., Frommer W.B., Eom J.S. (2018). SWEET11 and 15 as Key Players in Seed Filling in Rice. New Phytol..

[5] Wang S., Liu S., Wang J., Yokosho K., Zhou B., Yu Y.C., Liu Z., Frommer W.B., Ma J.F., Chen L.Q. (2020). Simultaneous Changes in Seed Size, Oil Content and Protein Content Driven by Selection of SWEET Homologues during Soybean Domestication. Natl. Sci. Rev..

[6] Doidy J., Grace E., Kühn C., Simon-Plas F., Casieri L., Wipf D. (2012). Sugar Transporters in Plants and in Their Interactions with Fungi. Trends Plant Sci..

[7] Wang S., Yokosho K., Guo R., Whelan J., Ruan Y.L., Ma J.F., Shou H. (2019). The Soybean Sugar Transporter *GmSWEET15* Mediates Sucrose Export from Endosperm to Early Embryo. Plant Physiol..

[8] Hu L., Zhang F., Song S., Yu X., Ren Y., Zhao X., Liu H., Liu G., Wang Y., He H. (2022). CsSWEET2, a Hexose Transporter from Cucumber (*Cucumis sativus* L.), Affects Sugar Metabolism and Improves Cold Tolerance in *Arabidopsis*. Int. J. Mol. Sci..

[9] Chen L.Q., Hou B.H., Lalonde S., Takanaga H., Hartung M.L., Qu X.Q., Guo W.J., Kim J.G., Underwood W., Chaudhuri B. (2010). Sugar Transporters for Intercellular Exchange and Nutrition of Pathogens. Nature.

[10] Chen H.Y., Huh J.H., Yu Y.C., Ho L.H., Chen L.Q., Tholl D., Frommer W.B., Guo W.J. (2015). The *Arabidopsis* Vacuolar Sugar Transporter SWEET2 Limits Carbon Sequestration from Roots and Restricts Pythium Infection. Plant J..

[11] Tao Y., Cheung L.S., Li S., Eom J.-S., Chen L.-Q., Xu Y., Perry K., Frommer W.B., Feng L. (2015). Structure of a Eukaryotic SWEET Transporter in a Homotrimeric Complex. Nature.

[12] Lauschke A., Maibaum L., Engel M., Eisengräber L., Bayer S., Hackel A., Kühn C. (2025). The Potato Sugar Transporter *SWEET1g* Affects Apoplasmic Sugar Ratio and Phloem-Mobile Tuber- and Flower-Inducing Signals. Plant Physiol..

[13] Valifard M., Le Hir R., Müller J., Scheuring D., Neuhaus H.E., Pommerrenig B. (2021). Vacuolar Fructose Transporter SWEET17 Is Critical for Root Development and Drought Tolerance. Plant Physiol..

[14] Klemens P.A.W., Patzke K., Deitmer J., Spinner L., Le Hir R., Bellini C., Bedu M., Chardon F., Krapp A., Neuhaus H.E. (2013). Overexpression of the Vacuolar Sugar Carrier *AtSWEET16* Modifies Germination, Growth, and Stress Tolerance in Arabidopsis. Plant Physiol..

[15] Wang L., Yao L., Hao X., Li N., Qian W., Yue C., Ding C., Zeng J., Yang Y., Wang X. (2018). Tea Plant SWEET Transporters: Expression Profiling, Sugar Transport, and the Involvement of CsSWEET16 in Modifying Cold Tolerance in Arabidopsis. Plant Mol. Biol..

[16] Zhang F., Li Z., Zhou J., Gu Y., Tan X. (2021). Comparative Study on Fruit Development and Oil Synthesis in Two Cultivars of *Camellia oleifera*. BMC Plant Biol..

[17] Lin P., Wang K., Wang Y., Hu Z., Yan C., Huang H., Ma X., Cao Y., Long W., Liu W. (2022). The Genome of Oil-*Camellia* and Population Genomics Analysis Provide Insights into Seed Oil Domestication. Genome Biol..

[18] Cao Y., Yao X., Ren H., Wang K. (2017). Determination of Fatty Acid Composition and Metallic Element Content of Four *Camellia* Species Used for Edible Oil Extraction in China. J. Consum. Prot. Food Saf..

[19] Hao L., Zhang X., Liu S. (2012). Risk Assessment to China’s Agricultural Drought Disaster in County Unit. Nat. Hazards.

[20] Lu K., Chen C., Zhou J., Yuan J., Lu M., Qiu J., Xiao Z., Tan X. (2025). Metagenomic and Metabolomic Profiling of Rhizosphere Microbiome Adaptation to Irrigation Gradients in *Camellia* Oil Trees. Ind. Crop. Prod..

[21] Qu X., Wang H., Chen M., Liao J., Yuan J., Niu G. (2019). Drought Stress–Induced Physiological and Metabolic Changes in Leaves of Two Oil Tea Cultivars. J. Am. Soc. Horticult. Sci..

[22] Guo P.R., Wu L.L., Wang Y., Liu D., Li J.A. (2023). Effects of Drought Stress on the Morphological Structure and Flower Organ Physiological Characteristics of *Camellia oleifera* Flower Buds. Plants.

[23] Chen Q., Hu T., Li X., Song C.-P., Zhu J.K., Chen L., Zhao Y. (2022). Phosphorylation of SWEET Sucrose Transporters Regulates Plant Root:Shoot Ratio under Drought. Nat. Plants.

[24] Du B., Cao Y., Zhou J., Chen Y., Ye Z., Huang Y., Zhao X., Zou X., Zhang L. (2024). Sugar Import Mediated by Sugar Transporters and Cell Wall Invertases for Seed Development in *Camellia oleifera*. Hortic. Res..

[25] Du B., Zou X., Wang Z., Zhang X., Cao Y., Zhang L. (2024). Genome-wide identification and expression analysis of the *SWEET* gene family in *Camellia oleifera*. Biotechnol. Bull..

[26] Cai H., Liang M., Qin X., Dong R., Wang X., Wang H., Sun S., Cui X., Yang W., Li R. (2025). Tonoplast Sugar Transporters Coordinately Regulate Tomato Fruit Development and Quality. Plant Commun..

[27] Xuan Y.H., Hu Y.B., Chen L.-Q., Sosso D., Ducat D.C., Hou B.-H., Frommer W.B. (2013). Functional Role of Oligomerization for Bacterial and Plant SWEET Sugar Transporter Family. Proc. Natl. Acad. Sci. USA.

[28] Nelson B.K., Cai X., Nebenfuehr A. (2007). A Multicolored Set of in Vivo Organelle Markers for Co-Localization Studies in Arabidopsis and Other Plants. Plant J..

[29] Singh J., Das S., Jagadis Gupta K., Ranjan A., Foyer C.H., Thakur J.K. (2023). Physiological Implications of SWEETs in Plants and Their Potential Applications in Improving Source–Sink Relationships for Enhanced Yield. Plant Biotechnol. J..

[30] Xu Y., Tao Y., Cheung L.S., Fan C., Chen L.-Q., Xu S., Perry K., Frommer W.B., Feng L. (2014). Structures of Bacterial Homologues of SWEET Transporters in Two Distinct Conformations. Nature.

[31] Gwon S., Park J., Huque A.K.M., Cheung L.S. (2023). The Arabidopsis SWEET1 and SWEET2 Uniporters Recognize Similar Substrates While Differing in Subcellular Localization. J. Biol. Chem..

[32] Gautam T., Dutta M., Jaiswal V., Zinta G., Gahlaut V., Kumar S. (2022). Emerging Roles of SWEET Sugar Transporters in Plant Development and Abiotic Stress Responses. Cells.

[33] Sosso D., Luo D., Li Q.-B., Sasse J., Yang J., Gendrot G., Suzuki M., Koch K.E., McCarty D.R., Chourey P.S. (2015). Seed Filling in Domesticated Maize and Rice Depends on SWEET-Mediated Hexose Transport. Nat. Genet..

[34] Ye Z., Du B., Zhou J., Cao Y., Zhang L. (2023). *Camellia oleifera* CoSWEET10 Is Crucial for Seed Development and Drought Resistance by Mediating Sugar Transport in Transgenic Arabidopsis. Plants.

[35] Breia R., Conde A., Badim H., Fortes A.M., Gerós H., Granell A. (2021). Plant SWEETs: From Sugar Transport to Plant–Pathogen Interaction and More Unexpected Physiological Roles. Plant Physiol..

[36] Valifard M., Khan A., Berg J., Le Hir R., Pommerrenig B., Neuhaus H.E., Keller I. (2024). Carbohydrate Distribution via SWEET17 Is Critical for Arabidopsis Inflorescence Branching under Drought. J. Exp. Bot..

[37] He Z., Zhang P., Jia H., Zhang S., Nishawy E., Sun X., Dai M. (2024). Regulatory Mechanisms and Breeding Strategies for Crop Drought Resistance. New Crop..

[38] Xing M.Q., Chen S.H., Zhang X.F., Xue H.W. (2023). Rice OsGA2ox9 Regulates Seed GA Metabolism and Dormancy. Plant Biotechnol. J..

[39] Mathan J., Singh A., Ranjan A. (2021). Sucrose Transport in Response to Drought and Salt Stress Involves ABA-Mediated Induction of *OsSWEET13* and *OsSWEET15* in Rice. Physiol. Plant..

[40] Zhang S., Wang H., Wang T., Zhang J., Liu W., Fang H., Zhang Z., Peng F., Chen X., Wang N. (2023). Abscisic Acid and Regulation of the Sugar Transporter Gene *MdSWEET9b* Promote Apple Sugar Accumulation. Plant Physiol..

[41] Kanno Y., Oikawa T., Chiba Y., Ishimaru Y., Shimizu T., Sano N., Koshiba T., Kamiya Y., Ueda M., Seo M. (2016). AtSWEET13 and AtSWEET14 Regulate Gibberellin-Mediated Physiological Processes. Nat. Commun..

[42] Morii M., Sugihara A., Takehara S., Kanno Y., Kawai K., Hobo T., Hattori M., Yoshimura H., Seo M., Ueguchi-Tanaka M. (2020). The Dual Function of OsSWEET3a as a Gibberellin and Glucose Transporter Is Important for Young ShootDevelopment in Rice. Plant Cell Physiol..

[43] Li S.F., Ye T.W., Xu X., Yuan D.Y., Xiao S.X. (2021). Callus Induction, Suspension Culture and Protoplast Isolation in *Camellia oleifera*. Sci. Hortic..

[44] Li S., Zhao R., Ye T., Guan R., Xu L., Ma X., Zhang J., Xiao S., Yuan D. (2022). Isolation, Purification and PEG-Mediated Transient Expression of Mesophyll Protoplasts in *Camellia oleifera*. Plant Methods.

[45] Zhang X., Feng C., Wang M., Li T., Liu X., Jiang J. (2021). Plasma Membrane-Localized SlSWEET7a and SlSWEET14 Regulate Sugar Transport and Storage in Tomato Fruits. Hortic. Res..

[46] Zhang W., Ruan C., Li J., Han P., Ding J., Liu L., Wu B., Ruan D. (2018). Screening of reference genes in four woody-oil trees and spatio-temporal expression analysis of *Actin* gene. Mol. Plant Breed..

[47] Zhou J., Du B., Chen Y., Cao Y., Yu M., Zhang L. (2022). Integrative Physiological and Transcriptomic Analysis Reveals the Transition Mechanism of Sugar Phloem Unloading Route in *Camellia oleifera* Fruit. Int. J. Mol. Sci..

