# Supplementary material for: Functional Studies and Expression Characteristics of the Vacuolar Sugar Transporter CoSWEET2a in Camellia oleifera
Source: Plants (Basel). 2025 Aug 22;14(17):2618. doi: 10.3390/plants14172618 (PMC12430227; doi:10.3390/plants14172618)
Supplement: Supplementary file 1 [file plants-14-02618-s001.zip › CoSWEET2a-plants-supplementary.pdf]

Table S1. Coding sequence (CDS) of *CoSWEET2a*

Gene name	CDS
CoSWEET2a	ATGACTCAAACTGTGTTGCTCTCCACTTACTCAGATTTCACTGATGCAGCTGGAATTGCTGGGAACCTCTTTGCTATTGTGC<br>TATTTGTGTCGCCCATACCCACATTCAGGAGAATTATCAGAAACAAGTCAACAGAACAGTTCTCAGGACTGCCATATATATA<br>TGGACTCTTGAATTGCTTGATATGCCTATGGTATGGCATGCCCATTGTATCTCCGGGTATTTTACCTGTTGCCACAGTCAACT<br>CGATTGGGGCGGTTTTCCAATTAATGTACCTAACCATCTTCATTATACATGCAGAGAACGCGAAAAAGGTGAAGATGTTGG<br>GACTCTTGCTAGTGGTTGTTGCTGCATTTGGTATTATAGCATTCATGAGCCTCACTTTATTTGAGCCTCCTAATCGACAGATC<br>TTCGTTGGATATTTGAGTGTCATTTCTCTTATATCCATGTTTGCTTCTCCGCTAGTTGTTATTAATTTGGTGATCAAAACAAAG<br>AGTGTTGAATTCATGCCGTTTTATCTCTCCCTTGCAACCTTTTTGATGAGTCTCTCTTTCTTTGCATTTGGAATGTTCAAGTG<br>CGATCCGTTCATTTATGTCCCAAATGGAATCGGAACAGTTCTGGGAATCGTACAGTTGGTGTTGTACTACTATTACAGCAAT<br>GCATATGGAAAAGCCTCAACAGAACCTTTGCTCGAGTCTTACGCGTGA

Table S2. Amino acid sequences used for homologous protein alignment and phylogenetic tree construction

Protein name	Scientific Name	Amino acid sequence
CoSWEET2a	*Camellia oleifera*	MTQTVLLSTYSDFTDAAGIAGNLFAIVLFVSPIPTFRRIIRNKSTEQFSGLPYIYGLLNCLICLWYGMPIVSPGILPVATVNSIGAVFQLMYLTIF<br>IIHAENAKKVKMLGLLLVVVAAFGIIAFMSLTLFEPPNRQIFVGYLSVISLISMFASPLVVINLVIKTKSVEFMPFYLSLATFLMSLSFFAFGMF<br>KCDPFIYVPNGIGTVLGIVQLVLYYYYSNAYGKASTEPLLESYA-
CsSWEET2a	*Camellia sinensis*	MVSLALSQISTIYKDAAGIAGNIFAFGLFVSPMPTFRRIIRNKSTEQFSGLPYIYALLNCMLSAWYGTPLISSDNVLVMTVNLIGAVFQLTYIT<br>LFITFTEKAKKLSMFGLLLAVFGLFAIVVAGSLHISDLSARRICIGFLSGAALISMFASPLLIIKLVIRTRSVEFMPFYLSLSTFLMSTAFFLYGIF<br>NFDPFIYVPNGIGAILGIVQLALFFYYKNTSGEASREPLLVTYT
atsweet2	*Arabidopsis thaliana*	MDVFAFNASLSMCKDVAGIAGNIFAFGLFVSPMPTFRRIMRNKSTEQFSGLPYIYALLNCLICLWYGTPFISHSNAMLMTVNSVGATFQLCY<br>IILFIMHTDKKNKMKMLGLLFVVFAVVGVIVAGSLQIPDQLTRWYFVGFLSCGSLVSMFASPLFVINLVIRTKSVEFMPFYLSLSTFLMSASF<br>LLYGLFNSDAFVYTPNGIGTILGIVQLALYCYYHRNSIEEETKEPLIVSYV
PdSWEET2	*Prunus dulcis*	MVFSASHSVFIICRDAAGIAGNIFAFGLFLSPIHTYRRIIRNRSTEEFSGLPYIYALLNCLICTWYGSPLVSSDNLLIMTVNSAGAVFQLVYIALF<br>IIYAEKSKKVRMLGFLLADFGLFAIIVFGSLQMTDLVMRRLIVGLLSCVSLISMFASPMFIINLVIRTKSVEFMPFYLSLSTFLMSTSFFLYGIFN<br>YDLFIYVPNGIGTILGIIQLALYFYYKDSSKEDSREPLMVPYP
ZjSWEET2	*Ziziphus jujuba* Mill.	MNLFVSNSVYIFLKNAAGVAGNIFAIGLFVSPIHTFRRILRNRSTEQFSGLPYIYALLNCLICLWYGMPIISPDNLMVLTVNSAGAVFQLVYIIL<br>FISYAEKSQKVRMLVLLLAVFGLFAIIVGGSLQIIDFSMRRMVVGLLSCASLISMFASPLFIINLVIRTRSVEFMPFYLSLSTFLMSTSFLLYGLF<br>NSDPFIFVPNGIGTILGIVQLALYFYYHNSSQEDSREPLLVSYA
AtSWEET1	*Arabidopsis thaliana*	MNIAHTIFGVFGNATALFLFLAPSITFKRIIKNKSTEQFSGIPYPMTLLNCLLSAWYGLPFVSKDNTLVSTINGTGAVIETVYVLIFLFYAPKKE<br>KIKIFGIFSCVLAVFATVALVSLFALQGNGRKLFCGLAATVFSIIMYASPLSIMRLVVKTKSVEFMPFFLSLFVFLCGTSWFVYGLIGRDPFVA<br>IPNGFGCALGTLQLILYFIYCGNKGEKSADAQKDEKSVEMKDDEKKQNVVNGKQDLQV
atsweet2	*Arabidopsis thaliana*	MDVFAFNASLSMCKDVAGIAGNIFAFGLFVSPMPTFRRIMRNKSTEQFSGLPYIYALLNCLICLWYGTPFISHSNAMLMTVNSVGATFQLCY<br>IILFIMHTDKKNKMKMLGLLFVVFAVVGVIVAGSLQIPDQLTRWYFVGFLSCGSLVSMFASPLFVINLVIRTKSVEFMPFYLSLSTFLMSASF<br>LLYGLFNSDAFVYTPNGIGTILGIVQLALYCYYHRNSIEEETKEPLIVSYV
AtSWEET3	*Arabidopsis thaliana*	MGDKLRLSIGILGNGASLLLYTAPIVTFSRVFKKKSTEEFSCFPYVMTLFNCLIYTWYGLPIVSHLWENLPLVTINGVGILLESIFIFIYFYYASP<br>KEKIKVGVTFVPVIVGFGLTTAISALVFDDHRHRKSFVGSVGLVASISMYGSPLVVMKKVIETRSVEYMPFYLSFFSFLASSLWLAYGLLSH

		DLFLASPNMVATPLGILQLILYFKYKNKKDLAPTTMVITKRNDHDDKNKATLEFVVDVDRNSDTNEKNSNNASSI
AtSWEET4	*Arabidopsis thaliana*	MVNATVARNIAGICGNVISLFLFLSPIPTFITIYKKKKVEEYKADPYLATVLNCALWVFYGLPMVQPDSLLVITINGTGLAIELVYLAIFFFFSP<br>TSRKVKVGLWLIGEMVFVGIVATCTLLLFHTHNQRSSFVGIFCVIFVSLMYIAPLTIMSKVIKTKSVKYMPFSLSLANFLNGVVWVIYALIKF<br>DLFILIGNGLGTVSGAVQLILYACYYKTTPKDDEDEEDEENLSKVNSQLQLSGNSGQAKRVSA
AtSWEET5	*Arabidopsis thaliana*	MTDPHTARTIVGIVGNVISFGLFCAPIPTMVKIWKMKSVSEFKPDPYVATVLNCMMWTFYGLPFVQPDSLLVITINGTGLFMELVYVTIFFV<br>FATSPVRRKITIAMVIEVIFMAVVIFCTMYFLHTTKQRSMLIGILCIVFNVIMYAAPLTVMKLVIKTKSVKYMPFFLSLANFMNGVVWVIYA<br>CLKFDPYILIPNGLGSLSGIIQLIIYITYYKTTNWNDDDEDKEKRYSNAGIELGQA
AtSWEET6	*Arabidopsis thaliana*	MVHEQLNLIRKIVGILGNFISLCLFLSPTPTFIHIVKKKSVEKYSPLPYLATLLNCLVRALYGLPMVHPDSTLLVTISGIGITIEIVFLTIFFVFCG<br>RQQHRLVISAVLTVQVVFVATLAVLVLTLEHTTDQRTISVGIVSCVFNAMMYASPLSVMKMVIKTKSLEFMPFLLSVVGFLNAGVWTIYGF<br>VPFDPFLAIPNGIGCVFGLVQLILYGTYYKSTKGIMEERKNRLGYVGEVGLSNAIAQTEPENIPYLNKRVSGV
AtSWEET7	*Arabidopsis thaliana*	MVFAHLNLLRKIVGIIGNFIALCLFLSPTPTFVRIVKKKSVEEYSPIPYLATLINCLVWVLYGLPTVHPDSTLVITINGTGILIEIVFLTIFFVYCG<br>RQKQRLIISAVIAAETAFIAILAVLVLTLQHTTEKRTMSVGIVCCVFNVMMYASPLSVMKMVIKTKSVEFMPFWLSVAGFLNAGVWTIYAL<br>MPFDPFMAIPNGIGCLFGLAQLILYGAYYKSTKRIMAERENQPGYVGLSSAIARTGSEKTANTNQEPNNV
AtSWEET8	*Arabidopsis thaliana*	MVDAKQVRFIIGVIGNVISFGLFAAPAKTFWRIFKKKSVEEFSYVPYVATVMNCMLWVFYGLPVVHKDSILVSTINGVGLVIELFYVGVYL<br>MYCGHKKNHRRNILGFLALEVILVVAIILITLFALKGDFVKQTFVGVICDVFNIAMYGAPSLAIIKVVKTKSVEYMPFLLSLVCFVNAGIWTT<br>YSLIFKIDYYVLASNGIGTFLALSQLIVYFMYYKSTPKEKTVKPSEVEISATERV
AtSWEET9	*Arabidopsis thaliana*	MFLKVHEIAFLFGLLGNIVSFGVFLSPVPTFYGIYKKKSSKGFQSIPYICALASATLLLYYGIMKTHAYLIISINTFGCFIEISYLFLYILYAPREA<br>KISTLKLIVICNIGGLGLLILLVNLLVPKQHRVSTVGWVCAAYSLAVFASPLSVMRKVIKTKSVEYMPFLLSLSLTLNAVMWFFYGLLIKDK<br>FIAMPNILGFLFGVAQMILYMMYQGSTKTDLPTENQLANKTDVNEVPIVAVELPDVGSDNVEGSVRPMK
AtSWEET10	*Arabidopsis thaliana*	MAISQAVLATVFGILGNIISFFVCLAPIPTFVRIYKRKSSEGYQSIPYVISLFSAMLWMYYAMIKKDAMMLITINSFAFVVQIVYISLFFFYAPK<br>KEKTLTVKFVLFVDVLGFGAIFVLTYFIIHANKRVQVLGYICMVFALSVFVAPLGIIRKVIKTKSAEFMPFGLSFFLTLSAVMWFFYGLLLKD<br>MNIALPNVLGFIFGVLQMILFLIYKKPGTKVLEPPGIKLQDISEHVVDVVRLSTMVCNSQMRTLVPQDSADMEATIDIDEKIKGDIEKNKDEK<br>EVFLISKN

AtSWEET11	*Arabidopsis thaliana*	MSLFNTENTWAFVFGLLGNLISFAVFLSPVPTFYRIWKKKTTEGFQSIPYVVALFSATLWLYYATQKKDVFLLVTINAFGCFIETIYISMFLA<br>YAPKPARMLTVKMLLLMNFGGFCAILLLCQFLVKGATRAKIIGGICVGFSVCVFAAPLSIIRTVIKTRSVEYMPFSLSLTLTISAVIWLLYGLA<br>LKDIYVAFPNVLGFALGALQMILYVVYKYCKTSPHLGEKEVEAAKLPEVSLDMLKLGTVSSPEPISVVRQANKCTCGNDRRAEIEDGQTPK<br>HGKQSSSAAAT
AtSWEET12	*Arabidopsis thaliana*	MALFDTHNTWAFVFGLLGNLISFAVFLSPVPTFYRICKKKTTEGFQSIPYVVALFSAMLWLYYATQKKDVFLLVTINSFGCFIETIYISIFVAF<br>ASKKARMLTVKLLLLMNFGGFCLILLLCQFLAKGTTRAKIIGGICVGFSVCVFAAPLSIIRTVIKTKSVEYMPFSLSLTLTISAVIWLLYGLAL<br>KDIYVAFPNVIGFVLGALQMILYVVYKYCKTPSDLVEKELEAAKLPEVSIDMVKLGTLTSPEPVAITVVRSVNTCNCNDRNAEIENGQGVR<br>NSAATT
AtSWEET13	*Arabidopsis thaliana*	MALTNNLWAFVFGILGNIISFVVFLAPVPTFVRICKKKSTEGFQSLPYVSALFSAMLWIYYAMQKDGTAFLLITINAFGCVIETIYIVLFVSYA<br>NKKTRISTLKVLGLLNFLGFAAIVLVCELLTKGSTREKVLGGICVGFSVSVFAAPLSIMRVVVRTRSVEFMPFSLSLFLTISAVTWLFYGLAIK<br>DFYVALPNVLGAFLGAVQMILYIIFKYYKTPVAQKTDKSKDVSDHSIDIAKLTTVIPGAVLDSAVHQPPALHNVPETKIQLTEVKSQNMTDP<br>KDQINKDVQKQSQV
AtSWEET14	*Arabidopsis thaliana*	MVLTHNVLAVTFGVLGNIISFIVFLAPVPTFVRICKKKSIEGFESLPYVSALFSAMLWIYYALQKDGAGFLLITINAVGCFIETIYIILFITYANK<br>KARISTLKVLGLLNFLGFAAIILVCELLTKGSNREKVLGGICVGFSVCVFAAPLSIMRVVIRTKSVEFMPFSLSLFLTISAITWLFYGLAIKDFY<br>VALPNILGAFLGAVQMILYVIFKYYKTPLVVDETEKPKTVSDHSINMVKLSSTPASGDLTVQPQTNPDVSHPIKTHGGDLEDQMDKKMPN
AtSWEET15	*Arabidopsis thaliana*	MGVMINHHFLAFIFGILGNVISFLVFLAPVPTFYRIYKRKSTESFQSLPYQVSLFSCMLWLYYALIKKDAFLLITINSFGCVVETLYIAMFFAY<br>ATREKRISAMKLFIAMNVAFFSLILMVTHFVVKTPPLQVSVLGWICVAISVSVFAAPLMIVARVIKTKSVEYMPFTLSFFLTISAVMWFAYGL<br>FLNDICIAIPNVVGFVLGLLQMVLYLVYRNSNEKPEKINSSEQQLKSIVVMSPLGVSEVHPVVTESVDPLSEAVHHEDLSKVTKVEEPSIENG<br>KCYVEATRPETV
AtSWEET16	*Arabidopsis thaliana*	MADLSFYVGVIGNVISVLVFLSPVETFWRIVQRRSTEEYECFPYICTLMSSSLWTYYGIVTPGEYLVSTVNGFGALAESIYVLIFLFFVPKSRF<br>LKTVVVVLALNVCFPVIAIAGTRTLFGDANSRSSSMGFICATLNIIMYGSPLSAIKTVVTTRSVQFMPFWLSFFLFLNGAIWGVYALLLHDMF<br>LLVPNGMGFFLGIMQLLIYAYYRNAEPIVEDEEGLIPNQPLLA
AtSWEET17	*Arabidopsis thaliana*	MAEASFYIGVIGNVISVLVFLSPVETFWKIVKRRSTEEYKSLPYICTLLGSSLWTYYGIVTPGEYLVSTVNGFGALVETIYVSLFLFYAPRHLK<br>LKTVDVEAMLNVFFPIAAIVATRSAFEDEKMRSQSIGFISAGLNIIMYGSPLSAMKTVVTTKSVKYMPFWLSFFLFLNGAIWAVYALLQHDV<br>FLLVPNGVGFVFGTMQLILYGIYRNAKPVGLSNGLSEIAQDEEEGLTSRVEPLLS

CsSWEET1a	*Camellia sinensis*	MGNTAHFVFGVFGNVNGLLLFLAPTITFKRIIMSKSTEQFSGIPYVMTLLNCLLSAWYGMPFVSPHNMLVSTINGTGAAIEAIYVFIFIIFAPK<br>KEKGKILGLLIFVLSVFTAVALISMFALHGKNRKIFCGLAASIFSIIMYASPLSIMRMVIKTKSVEFMPFFLSLFVFLCGTSWFVFGLLGKDPFV<br>AVPNGFGSGLGVIQLILYVIYRNNKGEAKKPPNADGSLEIGLEKSQPHEEKRSSYEEKRSSK
CsSWEET1b	*Camellia sinensis*	MGGVAHLVFGVFGNANGLLLFLAPTITFKRIIMNKSTERFSGIPYVMTLLNCLLSAWYGLPFVSPHNLLVTTINVTGAAIESIYVLIFLILSPRR<br>EKGKILGLLTVVLAIFAAVALISYFALHGRNRTIFCGLAASIFSIIMYASPLSIMRTVIRTKSVEYMPFFLSLFVFLCGTSWFLYGLLGKDPFIA<br>VPNGFGSGLGTVQLILYAIYHNKKGETKKPSVDGSVEMELAKPQQSQNGHV
CsSWEET2a	*Camellia sinensis*	MNFAASASVLSICKDAAGVAGNIFAFGLFVSPMPTFRRIIRNKSTEQFSGLPYIYALLNCMLSAWYGTPLISSDNVLVMTVNLIGAVFQLTYI<br>TLFITFTEKAKKLSMFGLLLAVFGLFAIVVAGSLHISDLSARRICIGFLSGAALISMFASPLLIIVPNGIGAILGIVQLALFFYYKNTSGEASREPL<br>LTDAFLMLEQLSS
CsSWEET2b	*Camellia sinensis*	MISLALSRTLTIWKDAAGIAGNIFAFGLFVSPIPTFRRIIRNKSTEQFSGLPYIYALLNCLICAWYGTPLVSLDNLFVTTVNSVGAVFQITYITM<br>FITYAEKENKLRMLGLLLADLSIFAIVLVGSLHLSDLTTRHIFIGFLSGASLVSMFASPLFVINVVIRTRSVEFMPFYLSLSTFLMSTSFFLYGIL<br>NGDPFICVPNGIGTILGIVQLALFFYYKNASGEVSREPLIISST
CsSWEET2c	*Camellia sinensis*	MISLALSRTLTIWKDAAGIAGNIFAFGLFVSPIPTFRRIIRNKSTEQFSGLPYIYALLNCLICAWYGTPLVSLDNLFVTTVNSVGAVFQITYITM<br>FITYAEKENKLRMLGLLLADLSIFAIVLVGSLHLSDLTTRHIFIGFLSGASLVSMFASPLFVINVVIRTRSVEFMPFYLSLSTFLMSTSFFLYGIL<br>NGDPFICVPNGIGTILGIVQLALFFYYKNASGEVSREPLIISST
CsSWEET3a	*Camellia sinensis*	MGDRLRLSVGVMGNVASMLLYSAPILTFVRVIKKKSNEDFSCVPYIIALLNCFLYTWYGLPVRKVAMITIPVILVFCITAIISAFAFHDHRHR<br>KIFVGSIGLVASIAMYGSPLVVVKKVIETKSVEFMPFYLSLFSFLASSLWMTYGLLGHDLFLAVCILQLVIYFKYRKRGIMGGPHKCDIEKNG<br>DKTEQQLQVMVAEDTNGKI
CsSWEET3b	*Camellia sinensis*	MGDRLRPAVGVMGNVASMLLYTAPILTFSRVIKKKSTEEYSCVPYIIALLNCFLYTWYGLPIVSYSWENFPLITINSLGLLLELSFILIYFCSLL<br>LKDNWLLCTYFYEKDINIYIKMAQKKVTMITIPVILIFCITPTISTFAFRDHHNRKVFVGTIGLVASVVMYDSPLVVVQVIRIKSVEFMPFYLS<br>FFSFLASLLWMAYRLLSHDLLLSPKLVGSPLGIVQLVLYFKYKKMAIINEPAKWNIEKNSEKTKQQLQLVTTEDTNGKI
CsSWEET3c	*Camellia sinensis*	MGDRLRPAVGVMGNVASMLLYTAPILTFSRVIKKKSTEEYSCVPYIIALLNCFLYTWYGLPIVSYSWENFPLITINSLGLLLELSSILIYFCSLL<br>LKDNWLLCTYFYEKDINIYIKMAQKKVTMITIPVILIFCITPTISTFAFRDHHNRKVFVGTIGLVASVVMYDSPLVVVQVIRTKSVEFMPFYLS<br>FFSFLASLLWMAYRLLSHDLLLSPKLVGSPLGIVQLVLYFKYKKMAIINEPAKWNIEKNSEKTKQQLQLVTTEDTNGKI

CsSWEET5a	*Camellia sinensis*	MVGTDGIRTIVGVIGNVISLFLFLSPVPTLFKICKSKSVQEFKPYPYVATVLNCAMWIFYGLPVVHPDSILVVTINGVGLLIEIVYVLIFLTYSN<br>WPKRKKILIALLVEVIFMAIVVFITMTFLHTTKARSTLVGLLCIVFNIIMYTSPLTVMRSVIKTKSVKFMPFYLSLANFCNGLVWLIYALLKFD<br>PYITIPNGLGALSGAIQLILYATYYKTTNWDDDGPLESSEVQLSNRNIP
CsSWEET5b	*Camellia sinensis*	MPTFWRIFKNKSVEEFKPDPYLATVVNCMFWVFYGLPIIHPDSTLVITINSVGLALELFYLTIFLIYTTNHKRLKIVLYLVLEVVVLVAVGVP<br>TILKVHTHEKRSMVVGIFCVIFGGLMYAAPLTIMRKVIKTKSVEFMPFWLSFAAFSNGVIWLIYALIKFDPYIFTGNGLGTLCGAAQLILYAY<br>YYKSTPKGNEKNPPQVQMNPLLHPPFFFFWVAWQFFIFLDFCRFYTYDGCFSLPVIGFIKIILFEFSFLLSAISMAATLSCHYCPSLSPSSKLINL<br>YNIPRLCPLLKFLENPLFSPTQTLLFFPNSPSISNPDQEDRVFPFKECAISIALAIGLITGVPALDWCNYANAATPAVLPELSGLISGPPIQDPRA<br>LLRHALPIDNKAIREVQKPLEDITDSLKIAGLKALDSVERNVRQASRALKQGKSLLVSGLAESKKDHGIELLGNLEDGMGELQI
CsSWEET5c	*Camellia sinensis*	MEPETIRTTVGIIGNVISFFLFLSPVPTFFKIIKAKSVQEFKADPYVATVLNCALWVIYGLPFVHPDSLLVITINGTGLVIELAYVLIFITYSDWQ<br>KRRKIILALFLEIIFVVFIFFITMTFLHTTQARSMLIGILCLVFNIVMYTAPLTVMKLVIQTKSVKYMPFFLSVANFANGCVWLVYALLKFDPY<br>IAIPNGLGTLSGAIQLILYATYYKTTNWDEDDDTPAEISDVELSKKNSTL
CsSWEET7a	*Camellia sinensis*	MAAVNAARTVIGVIEMFIGMNANVILKLLIYDAGVVKLKLTIVRIVKNKSVEEFEPDPYIAAVMNCICWVYYALPFIHPDSMLVLSINSVGL<br>ALELGYLVIFLIYTNNNKQRLKIIVCLGIEAVITGVIAAISLTKFHTHASRSIFVGIFSVIFGVILYSSPLTIMRKVIQTKSVEFMPFWLSVAAFSN<br>GLIWTIYASLKFDPFIMTGNSLGALLGAAQLILYWYYHKSTPKADQKKPCEVQLPEICLSPLVIGKSANMPAYSVGNLGLSFDTDYLQSIPM<br>AALISYHFCSSLINPNFSTNSPKNASASFRLRCSSESPRRFQLHKNQRDRLFPLKECAISVALAIGLITGVPALDWSYYYANAATPALPDLSVL<br>ISGPPIKDPGALLRYALPVDNKAIREVQKPLEDITDSLKIAGVKALDSVERNLRQASRALKQGKSMIVSGLAESKKEHGVELLDKIEAGMGE<br>LQQIVEDRNRDAVAPKQKELLNYVGGVEEDMVDGFPYEVPEEYQNMPLLKGRATVDMKVKVKDNPNVDECVFRIVLDGYNAPVTAGNF<br>IDLVERHFYDGMEIQRADGFVVQTGDPEGPAEGFIDPSTEKTRTIPLEIMVDGEKAPFYGETLEEPWALIVTFYASESTELGLYKAQTKLPFN<br>AFGTMAMARDEFEDNSASSQVFWLLKESELTPSNANILDGRYAVFGYVTENADLLADVKVGDVIESIQVVSGLDNLVNPSYKIAA
CsSWEET7b	*Camellia sinensis*	MFTCIHINIFSHINNYGNIISIILFMSPVPTFVQICKKGSVEQYSAAPYLATLINCGLWVLYGLPMVHPHSLLVITINGSGLVIELVYLLLFLTYT<br>DRKKRLRVLLIILGEFVVLGALGVLVMTLAHTTKLRSLIVGSVAMLGNVMMYASPLSVMRMVISTKSVEYMPLTLSLASCANGICWTTYA<br>LIRFDPFLAAPNGLGTLFGLAQLILYATFYKSTKKQMLESQGKGIEMGLAEPTNSAEEAEKISTAASEIHRT
CsSWEET7c	*Camellia sinensis*	MFTCIHINIFSHINNYGNIISIILFMSPVPTFVQICKKGSVEQYSAAPYLATLINCGLWVLYGLPMVHPHSLLVITINGSGLVIELVYLLLFLTYT<br>DRKKRLRVLLIILGEFVVLGALGVLVMTLAHTTKLRSLIVGSVAMLGNVMMYASPLSVMRMVISTKSVEYMPLTLSLASCANGICWTTYA<br>LIRFDPFLAAPNGLGTLFGLAQLILYATFYKSTKKQMLEKQGKGIEMGLAEPTNSGEEAEKISTAASEIHRT

CsSWEET9a	*Camellia sinensis*	MAILTASHMASAFGIFGNIVSFFVYLAPLAMLLLYYGLLKTNGVMIITINTIGCAFEATYLLVFMIYATREAKLLIMFSVRAFGLITLCTFLFSKGHQRVTIVGWICVVFSVSVFALLSIMRLVIKTKSVEYMPFAHSLFLTLCAIMWFFYGFLIKDYNIATPNILGFAFGIAQMALYIVYKDTKKQVLPEVKLQDLGTVIDLSAIEMEQNFKADTDQMPKEIIVVDEDGDNNKSIKPSDSNV
CsSWEET9b	*Camellia sinensis*	MAILTASHMASAFGILGNIVSFFVYLAPLPTFYRIYKKKSTEGFQSIPYAVALFSAMLLLYYGFLKTNGLMIITINTVGCAIEATYLLVFMIYATREAKIYTTKLLIMFNVGAFGLITLCTFLFSKGHQRVTIVGWICAVFSVSVFAAPLSIMRLVIKTKSVEYMPFALSFFLTLCAIMWFFYGFLIKDYYIATPNILGFAFGIAQMALYIVYKDTKKQVLPVVKLQDLGTVIDLSAIEMEQNFKADTDQMPKEIIAVDEDGDNNKSIKPSESNV
CsSWEET10a	*Camellia sinensis*	MGGFSSHNLAFAFGLLGNIISFFVFLSPIKNQPKGFQSVPYIVALFSAMLLMYYAFLKKNDSTLIITINSFGCLVEIIYICVYLFYAPKKIKFKTVKLIGLMLGVFGAILILTQFLVKKSSTRFHIVGWICLVLSVSVFAAPLCILKQVIRTKSVEFMPFSLSLSLTLNAIMWFFYGLLIKDFNIAIPNVLGFIFGIIQMVLYAIYKNPKKVVKDQKFSSDQIPNEVIALEEEKLSELVEQVIDVVKLSSIACQEIGPVMLHLNGHNIARELNIVKPNLEVTVPV
CsSWEET10b	*Camellia sinensis*	MYYALLNSNETLLITINSFGCLIESFYIGVHLFYAPKKVKAVSLLLLGTGWFGLIFILTNFLTRGQARLHIVGWFCLVFSVCVFAAPLNIMNQVIRTKSVEFMPFYLSLSLTFSAIMWFFYGILRKDLNISIPNVIGFILGVLQMILYAIYKNGKKVEEQGLHVVEERQTEVVEEAVEQCIDVSAKV
CsSWEET10c	*Camellia sinensis*	MGGFSSHNLAFAFGLLGNIISFFVFLSPLPTFYQIFKKKSTEGFQSVPYIVALFSAMLLMYYAFLKKIDSTLIITINSFGCLVETIYICVYLFYAPKKIKFKTMKLLGFMLGGFGAILILTQFLIKKSSTRFHIVGWICLLFSVSVFAAPLCILKQVIRTKSVEFMPFSLSLSLTLNAVMWFFYGLLIKDFNIAIPNVLGFIFGIIQMVLYAIYKNPKKVVAKDQKFSSDQIPNKVIALEEEKLSELVEQVIDVVKDQKFSSDQIPNKVIALEEEKLSELVEQVIDVVKLSSIACQEIIPVMPHLNGHNIAKELNTIKPNLEVTVPV
CsSWEET12	*Camellia sinensis*	MITNNFNKNNISRTGILESTLCSITSVLVYLSPISTFYKIYKKKSSQGFQSLPYVVALLSAALWVYYAILNSLDIILMTISSFGCLIEYFYIGVHLFYAPKKVRSVSLLLLGIGGIGLIFILTNFLTKGQPRLQIVGWFCVVFSVCTYAAPLNIMKQVIQTKNVEFMPFYLSLSLTISAIMWFFYGILTKNFIIYIPNVIGSILGVLQMILYAIYKNGKKVEEQGLHVVEERQTEVAEKAVEQCIDVSAEV
CsSWEET15a	*Camellia sinensis*	MEIFGVHHPLVFTFGILGNIVSIFVYLAPLPTFIEICRKKSTLGFQSLPYVVALFSATLWMYYALLKPDAIPLISINSFGCIVETIYIIIFLAYASKKAKNHTAKLLASMNVGLFSVILLITQLLIKESFRVSVIGWICVAISVIVFAAPLSIVFHVVKTRSVEFMPFTLSFFLTLSAIMWFGLWPTKKGLLPNIFGFLLGLLQMLLYGIYRNAKEVVKEIKLPEHIISIVIDDDNDGEEGEKHDQTDHNHEKSVDLATHERSQAKYAA
CsSWEET15b	*Camellia sinensis*	MAILTASHMASAFGILGNIVSFFVYLAPLSTFYRIYKKKSTEGFQSTPYAVALFSAMLLLYYGFLKTNGLMIITINTIGCAVEATYLLVFMIYATREAKIYTTKLLIMFNVGAFGLITLCTFLFSKGHQQVTIVGWICAVFFVSVFAAPLSIMVLRLVIKTKSVEYMPFALSFFLTLCAIMWFLYGFFIKDYYIAVNADAFGIAQMALYIVYKYAKKQILSQIQIQLKLFGLVSFHFLGRGRRVFLMSESPSFAIIFFYNDPKEIIVVDEDGDNKKSINSSESNV

CsSWEET15c	*Camellia sinensis*	MAILTASHMASAFGILGNIVSFFVYLAPLSTFYRIYKKKSTEGFQSTPYAVALFSAMLLLYHGFLKTNGLMIITINTIGCAVEVTYLLVFMIYA TREAKIYTTKLLIMFNVGAFGLIALCTFLFSKGHQRVTIVGWICAVFSVSVFAAPLSIMRLVIKTKSVEYMPFAFSFFLTLCAIIWFLYGFFIKD YYIEIPNILGFAFGIAQMALYIVYKYAKKQISPQVKPQDLGTVINLVMLI
CsSWEET17a	*Camellia sinensis*	MASLSFIIGIIGNIISILVFASPIGTFKGVVKKKSTENFKALPYITTLLSTSLWTFYGILKPGGLLILTVNGAGAVLQFIYVTLFLIYAPRDIKVKS MKLVAILDVGFLGAVIAVTMLTIHGSLKLTFVGILCAALTIGMYAAPLAVMRTVINTTSVEYMPFFLSFFLFLNAGVWSVYALLVKDIFIGV PNAIGFILGSAQLILYLVYKNKSSSAKSKDEMEEEEEEGSAHLVKTSIEMQDLDDHDDLKSTNRNLNKGRSLPKPLVSRQYSINKIMKTFSLH PYELNSGSLHENDVENGSTKDHP
CsSWEET17b	*Camellia sinensis*	MEGLSFFVGVIGNIVSVLVFLSPIGTFWRVIKHRSTEEFESLPYICTLLNSSLWTYYGITKPGEILVATVNGFGIVVEAVYVTLFLIYAPKGMR TAILVGILDVGLLAVAIAVTQLAMNRDVRIDAIGFMGAALNIIMYASPLAAMKTVVTTKSVEYMPFFLSFTLFLNGGIWAFYAVLVHDLFL GVPNGTGFILGIAQLVLYAIYRNAKPTRNTTFDVLEEGCSQHEHLIPSSHQFIS
CsSWEET17c	*Camellia sinensis*	MKLVAIFDVGFLGAVIAVTLLAIHGSLRFTFVGILCAALTIGMYAAPLAVMRIVIKTASVEYMPFFLSFFLFLNAGVWSVYAFLVKDFFVGV PNAIGFVLGSAQLILYLVYKNKSSSAKSEDEMEDEEKEGPAHLVKTSIEMHGLDDHLKNKNRNLKKGRSLPNPPVSRQYSINKIMKTFSLRP YELNSGSLHVNDVENGSTKDHP

Table S3. Sequences of primers used in this study

Purpose	Primers name	Primer sequence (5'-3')
Gene clone	CoSWEET2a	F：ATGGGTGGGTTCTCTAGTCACAAT<br>R：TTAGACCGGCACGGTGACT
Subcellular localization	CoSWEET2a-pCAMBIA1300	F：GGGGCCCGGGGTCGACATGGGTGGGTTCTCTAG<br>R：CCATGGTACCGGATCCGACCGGCACGGTGAC
BiFC	CoSWEET2a-cYFP	F：CGCCACTAGTGGATCCATGACTCAAACTGTGT<br>R：TCCCGGGAGCGGTACCCGCGTAAGACTCGAGC
	CoSWEET2a-nYFP	F：CGCCACTAGTGGATCCATGACTCAAACTGTGT<br>R：TCCCGGGAGCGGTACCCGCGTAAGACTCGAGC
Identification of *Arabidopsis* mutants	atsweet2	LP：TCCATGTGTAAAGATGTTGCCGGA<br>RP：TCCGAATCACTAGGTTCTGCAAAG<br>LBal：ATTTTGCCGATTTCGGAAC
Identification of *Arabidopsis* restoration lines	CoSWEET2a-R	F：CCAACCACGTCTTCAAAGCA<br>R：CGGTGGTGCAGATGAACTTC
Quantitative Real-time PCR	qPCR-CoSWEET2a	F：GCAGAGAACGCGAAAAAGGT<br>R：ATATCCAACGAAGATCTGTCGATT
	qPCR-CoActin	F：AAACTACGGTTGCGGATAGAG<br>R：CTCCGGTGCATCCTTCATAAT

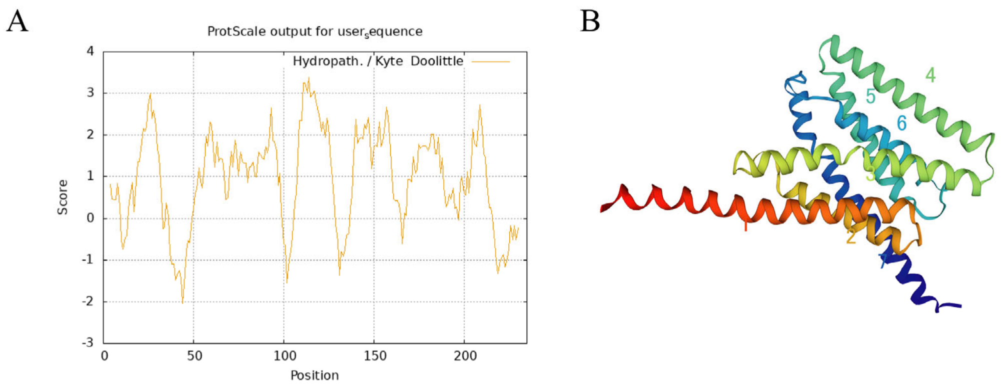

Figure S1. Hydrophilicity analysis (A) and tertiary structure prediction (B) of the CoSWEET2a protein.

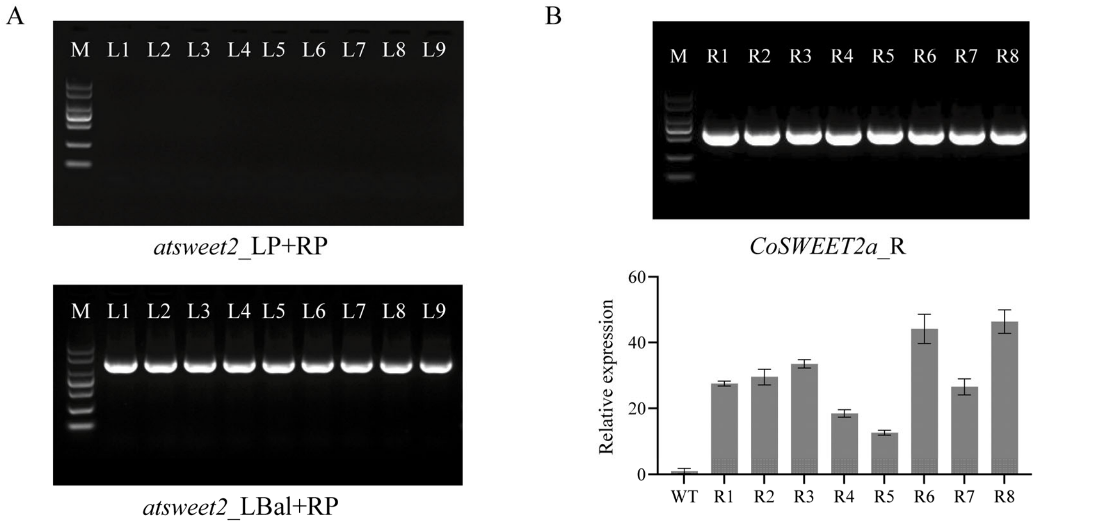

Figure S2. Identification of mutant and restoration lines of *Arabidopsis*. (A) Identification of homozygous *atsweet2* mutant lines in *Arabidopsis*. (B) Identification and relative expression analysis of *CoSWEET2a* restoration lines in *Arabidopsis*. M represents the DL2000 Marker.The expression data of WT was normalized to 1and relative to *Actin*. The data are shown as the means ± SDs of three replicates.

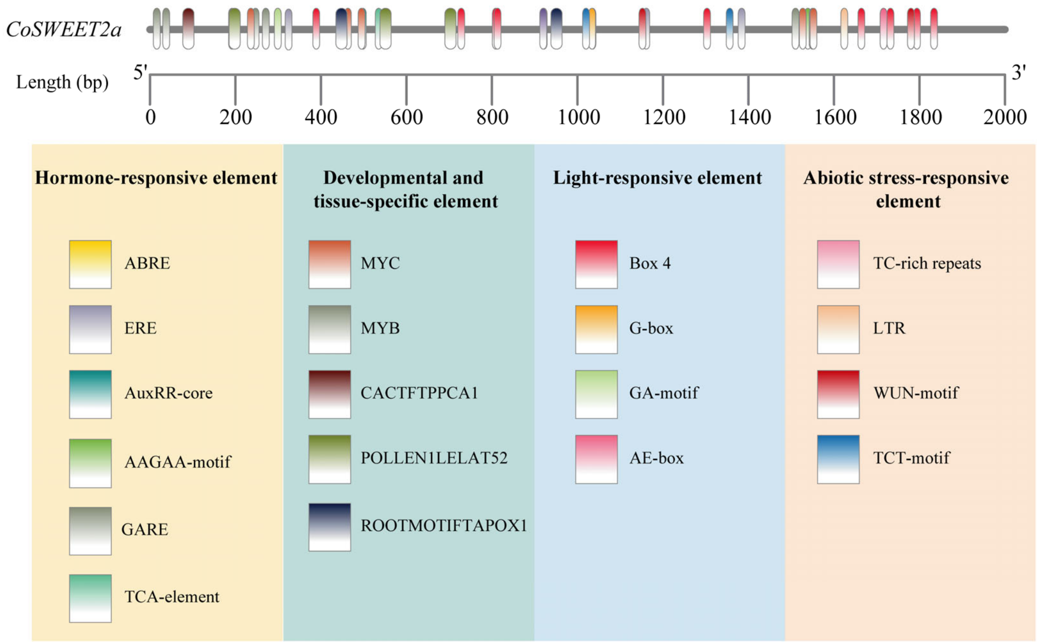

Figure S3. Analysis of cis-acting elements in the promoter region of the *CoSWEET2a* gene.
